# How single-cell immunology is benefiting from microfluidic technologies

**DOI:** 10.1038/s41378-020-0140-8

**Published:** 2020-07-13

**Authors:** Fabien C. Jammes, Sebastian J. Maerkl

**Affiliations:** Institute of Bioengineering, School of Engineering, École Polytechnique Fédérale de Lausanne, Lausanne, Switzerland

**Keywords:** Engineering, Nanoscience and technology

## Abstract

The immune system is a complex network of specialized cells that work in concert to protect against invading pathogens and tissue damage. Imbalances in this network often result in excessive or absent immune responses leading to allergies, autoimmune diseases, and cancer. Many of the mechanisms and their regulation remain poorly understood. Immune cells are highly diverse, and an immune response is the result of a large number of molecular and cellular interactions both in time and space. Conventional bulk methods are often prone to miss important details by returning population-averaged results. There is a need in immunology to measure single cells and to study the dynamic interplay of immune cells with their environment. Advances in the fields of microsystems and microengineering gave rise to the field of microfluidics and its application to biology. Microfluidic systems enable the precise control of small volumes in the femto- to nanoliter range. By controlling device geometries, surface chemistry, and flow behavior, microfluidics can create a precisely defined microenvironment for single-cell studies with spatio-temporal control. These features are highly desirable for single-cell analysis and have made microfluidic devices useful tools for studying complex immune systems. In addition, microfluidic devices can achieve high-throughput measurements, enabling in-depth studies of complex systems. Microfluidics has been used in a large panel of biological applications, ranging from single-cell genomics, cell signaling and dynamics to cell–cell interaction and cell migration studies. In this review, we give an overview of state-of-the-art microfluidic techniques, their application to single-cell immunology, their advantages and drawbacks, and provide an outlook for the future of single-cell technologies in research and medicine.

## Introduction

### Microfluidics

Microfluidics, the science of manipulating fluids on the microscale, has had considerable impact on biology, both in research and industry. Microfluidics significantly evolved over several decades and it is now used in almost all biological fields including biochemistry, cell signaling, drug testing, genomics, and proteomics. This success can be explained by various advantages microfluidic based approaches have over conventional technologies such as precise spatio- and temporal control of extremely small volumes, reducing costs and required sample volumes, while providing sensitivity and throughput.

The immune system is a complex system consisting of a variety of cell types that work in synergy to protect against invading pathogens and control infected and mutated cells. In addition to the diversity of cell types constituting the immune system, each cell type can present distinct features or can be genetically unique. For instance, macrophages can present several different phenotypes, ranging from anti- to pro-inflammatory. Cells from the adaptive immune system (T and B cells mostly) are genetically unique due to VDJ recombination and clonal selection. These unique features are often missed by conventional, bulk measurements that usually provide population level averages. Single-cell technologies present considerable advantages that can overcome the limitations of bulk measurements and can help achieve a better understanding of immune mechanisms, which in turn is expected to lead to efficient, personalized immune treatments for complex illness like autoimmune diseases and cancer.

In this review, we explore the growing field of microfluidics and present an up-to-date overview of the different approaches and techniques used for single-cell applications in immunology. We will briefly present the origins of microfluidics, focusing on the advantages this technology can bring to single-cell analyses and why reaching single-cell resolution is often a necessity in the study of the immune system. We will then briefly explain how microfluidics can be used to separate immune cell populations into more defined subsets; as the first necessary step to enable single-cell analyses. Finally, our main focus will be microfluidic techniques used in research to manipulate and study single immune cells covering active, passive, and droplet microfluidics.

#### Origins of microfluidics

Microfluidics originated in the microelectronics industry. In the late twentieth century, the field of microelectronics advanced with improved silicon-based micromachining and photolithography techniques. While microelectronics dates back to the 1970s, it was not until the 1990s that microfluidic devices started to be developed and applied to biological applications. With advances in liquid and gas chromatography, microfluidic devices were first used for biological separation using electrophoresis. For instance, Woolley et al.^1^ developed a microfluidic capillary gel electrophoresis system for DNA analysis with a considerable decrease in separation time. Since then, microfluidic techniques and devices are now used in a variety of biological applications covered in detailed reviews^2–5^.

#### Unique properties of microfluidic systems

Microfluidic technologies allow for the manipulation of fluids down to the micron and sometimes nanometer length scale and femto to microliter volumes. This entails several advantages that we briefly tabulate below as a non-exhaustive list.

- Volume reduction: Biological reactions often require expensive and/or rare molecules/cells. Compared to conventional methods, microfluidics can achieve orders of magnitude reductions in volumes.
- Laminar flow: One key aspect of the microfluidic world is the laminar flow regime that prevails at these length-scales. Low Reynolds number flows reduce flow dispersion and allow for precise control of flow behavior. Mixing of molecules between solutions is mostly due to diffusion at this length scale and therefore can be controlled by design.
- Parallelization: Microfluidic experiments can often be parallelized and multiplexed. By enabling spatial and temporal control of fluids, microfluidic devices can run several parallel biological reactions simultaneously.
- Integration and automation: Microfluidic devices are often made of materials with appealing properties (e.g. gas permeable, transparent, elastic), that are compatible with readouts such as fluorescence microscopy and can even incorporate microelectrodes for bio-electrical applications. In addition, on-chip fluid shunting can be computer controlled in order to create automated systems.

#### Materials used in microfluidics

In the early days of microfluidics, channels were directly etched into silicon wafers. However, glass soon became the new standard as it presented several advantages over silicon. Glass is relatively cheap, resistant, and transparent with good optical properties, making it a suitable material for biological applications involving microscopy^3^. However, at the beginning of the twenty-first century, another material quickly prevailed over glass and silicon^6^, namely silicone. Polydimethylsiloxane (PDMS) is an elastomeric polymer with excellent properties for microfluidics. PDMS, like glass, is transparent but also permeable to gases unlike glass and silicon. PDMS can be poured on a mold containing features on a silicon wafer, cured, peeled off the mold and bonded to another material such as glass. The master mold can be reused many times. This molding process drastically simplified the process of making microfluidic devices and reduced fabrication costs considerably. PDMS is therefore a suitable material candidate due to its inherent properties as an elastomeric, transparent polymer but also because of its molding microfabrication process that allows for fast, cost-efficient prototyping^7^. PDMS, while being one of the most commonly used materials, is not the only polymer used for microfluidic applications. Polymethylmethacrylate (PMMA) and Polycarbonate (PC) are also good candidates and typically used with injection molding methods for chip fabrication, albeit these two materials are not elastomeric. Glass and silicon are still used for certain applications as they do present specific advantages over polymers (e.g. better thermal resistance and conductivity).

### Immunology and single-cell analysis

As most bulk measurements obtain the average phenotype of a population of cells, they advanced the field of immunology and immunoengineering to a great extent: production of specific antibodies in large-scale, detection of viruses in blood samples, analyses of vaccine-induced T cells, etc. However, conventional technologies are inefficient in handling rare subsets of cells (such as antigen-specific T cells or B cells) because of their requirements for relatively large volumes and, as mentioned earlier, their inherent property of giving an average response of a population rather than returning specific, individual cell characteristics. Especially in the case of lymphocytes, which are genetically unique, being able to specifically measure single-cell phenotypes is required for advanced immunologic treatments such as for autoimmune disease treatments and cancer immunotherapy. Single-cell measurements are difficult to achieve with conventional techniques, hence the need for novel single-cell analysis tools in immunology. As a prominent example of a single-cell measurement, we will examine flow cytometry. Flow cytometry and more specifically fluorescence-activated cell sorting (FACS) have been incredibly useful tools in biology. Those technologies have allowed the detection and enrichment of specific immune populations in samples (e.g. isolate CD4^+^ or CD8^+^ T cells among a tumor sample). However, in FACS, each cell is only “a dot in a plot”. Features like affinities, secretion dynamics, and cell–cell interactions over time are not easily attainable by FACS. Recent advances enhanced the optical resolution of flow cytometry, such as polychromatic flow cytometry (PFC)^8^ and imaging flow cytometry^9^, but these technologies still lack temporal resolution for single-cell analysis. Microfluidic techniques make this additional dimension now accessible. We will describe how microfluidics can address single-cell analyses for immune cells and how this technology already had an impact on the field of immunology. We would like to mention a review by Chattopadhyay et al.^10^ on single-cell technologies for monitoring immune cells, which covers the shortcomings of bulk measurements and the need of single-cell resolution techniques.

### Enriching immune cell subpopulations

Although the focus of this review is on single-cell techniques for immune research, we believe that a short description of microfluidic techniques used to sort entire immune populations and enrich specific subsets can be useful as it is generally the first necessary step to obtaining single-cell measurements.

Microfluidic devices often use mechanical filters for sorting cell populations based on their physical properties (size, shape, etc.) in a label-free manner. As extensively described by Gosset et al.^11^, mechanical filters come in different shapes including weir-style filters, where a small planar gap is created allowing small cells to pass while retaining large cells. These type of filters have been used to filter white blood cells (WBCs) from whole blood samples^12^. Another type of sorting relies on pillar arrays to divert a cell’s flow path based on its size or trap large cells while letting small ones pass. This technique was used by Jiang and Aceto to extract WBCs and circulating tumor cells (CTCs) from whole blood samples^13,14^. More recently, groups including Chronis et al.^15^ used similar approaches for trapping WBCs. Arai and his group used pillar arrays to isolate and detect T and B cells from whole blood^16,17^ with specifically designed pillars of various geometries.

Another technique for filtering cells under constant flow is called hydrodynamic filtration. This technique relies on the laminar flow regime and specific channel geometries^11,18,19^ to achieve cell filtration in a passive, constant flow^11^. Another hydrodynamic method to separate cells based on their size is pinched flow fractionation^20,21^. The technique uses different inlets to pinch the sample flow against the microfluidic wall with another flow. In the process, small particles reach flow profiles closer to the wall than large particles. The pinched segment is then followed by a sudden widening of the channel with several branches. The particles follow the streamlines they were forced into during the pinching and the streamlines are then separated into the different branches. Therefore, large particles will end up in a different branch than small ones. Another technique relying on hydrodynamic filtration is called deterministic lateral displacement (DLD)^11^ and uses micropillar arrays. DLD allows for separation of particles based on their size. Small particles will flow through a micropillar array with their streamlines unchanged and therefore will follow a straight course. The larger the particle gets, the more likely it will hit a pillar and its course will change into a different streamline. Therefore, a correlation is observed between the size of the particle and the angle of its final streamline compared to the original one^11^. DLD was successfully used to filter whole blood samples and to separate WBCs from red blood cells (RBCs) and platelets^22,23^.

### Single immune cell analyses using microfluidics

As described above, microfluidic devices are useful tools to filter and sort subpopulations of cells, often without the need of fluorescent tags as required by FACS. In addition, microfluidics is ideally suited to study single cells. We will describe how different research groups over the years have used microfluidic techniques to study immune cells on the single-cell level. We will divide those techniques into three main microfluidic categories: passive devices, active devices, and droplet microfluidics. For each category, we will describe how these technologies were used in biological applications such as genomics, proteomics, and cell–cell/cell–environment interactions.

In this review, we will not cover microfluidic devices that use integrated electrodes, rely on electrophoresis, magnetophoresis, or other more specialized techniques. Our list is not exhaustive and there are several excellent reviews that have been published on microfluidic systems applied to single-cell analysis, including immune cells. We recommend several technical reviews describing single-cell techniques^24–28^. Our focus here is on single-cell microfluidic techniques for immune applications, this topic has also been described by excellent reviews. We strongly suggest our readers to complement their readings with those reviews^10,29–33^.

## Passive microfluidic devices

Passive microfluidic devices do not rely on active on-chip control elements for flow handling and regulate flow behavior by their inherent design. Fluids are driven through such chips either by capillarity or by applying a pressure source. Flow rates can be tuned by adjusting the design of the microfluidic chip or by adjusting the pressure source. Microwell and microtrap arrays are common approaches to isolate individual cells. Although glass microwell arrays generated by microengraving glass substrates have been around for a long time, one of the earliest uses of microwells in PDMS for single-cell isolation was performed by Rettig et al.^34^ in 2005. Similarly, microtrap arrays are often used to isolate individual cells for different applications. The works of Di Carlo et al.^35^ in 2005 and Faley et al.^36^ in 2009 are early examples of hydrodynamic cell traps for cell applications.

### Genomics

**Fig. 1:**
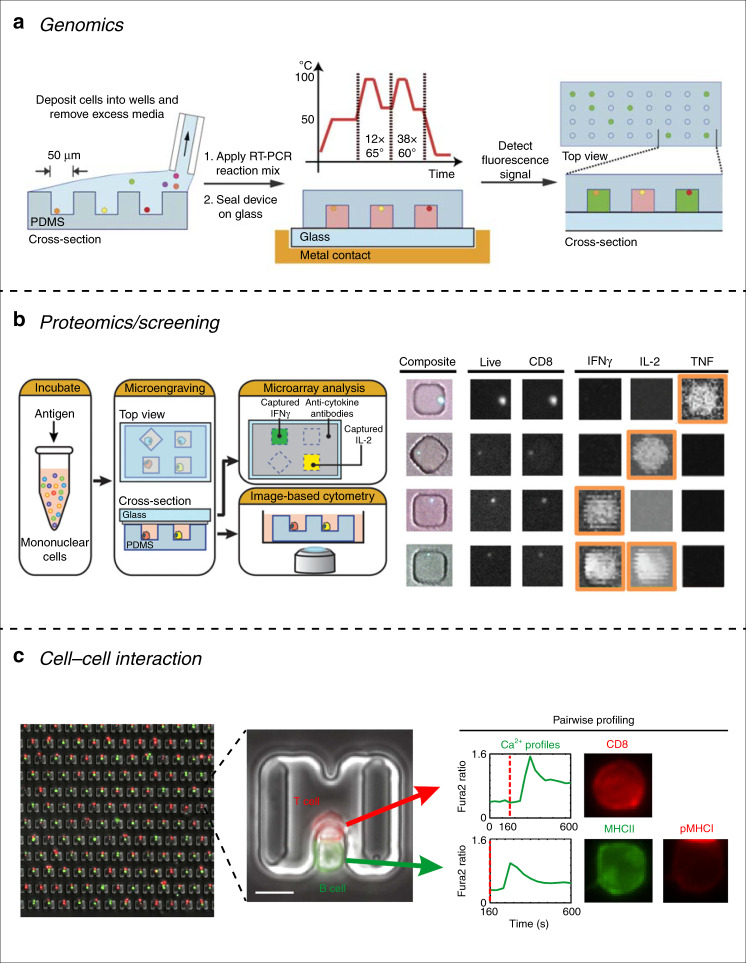
Passive devices for single-cell analysis. **a** Single cells can be trapped in microwells that can be sealed with RT-PCR solution and thermocycling will trigger the amplification reaction. **b** Microengraving is a useful tool for studying immune cell secretion at the single-cell level. **c** Interactions between immune cells can be studied at the single-cell level by trapping cell pairs using hydrodynamic traps. Reprinted with permission^38,45,53^

The ability to measure the transcriptome of individual cells is highly desirable, especially for immune research. One key aspect to better understand the immune system is to unravel the heterogeneity of immune cells. Adaptive immune cells are genetically unique. But in addition, each immune cell (both innate and adaptive) is at a given time and space polarized. Immune cells can be in a pro- or anti-inflammatory state; they can be idle or activated. The ability to identify the states of single immune cells is of great importance to understand an immune response. Single-cell RNA-sequencing (scRNA-seq) technologies have allowed a breakthrough in this respect^37^. Although scRNA-seq can be performed in “bulk” with the use of small tubes and vials, this technology requires precise control of small volumes down to the picoliter scale. Requirements that microfluidics can achieve and therefore represents a good candidate for scRNA-seq technologies for immune cell studies. Several groups implemented different approaches to isolate individual cells for scRNA-seq. An early example was the work of Love’s group^38^, where they developed a microfluidic platform with a microwell array of subnanoliter wells. Their platform was used to isolate individual B cells in microwells, filled with a RT-PCR solution before the entire chip was sealed to a glass slide. The amplification was performed after thermal lysis on a thermocycler (Fig. 1a). This technique was then combined with image-based cytometry and another process pioneered by Love’s group, called microengraving, which enables the capture and quantification of single-cell secretomes (we will explain this process just below). While not providing a quantitative gene expression platform, the approach of Gong et al. provided insights into the relationship between target gene transcription and protein expression/secretion levels. Other groups used microwell arrays to perform scRNA-seq^39,40^. Although Yuan and Sims did not focus on immune cells but rather on gliomas, the microfluidic platform they developed is of interest for immune applications. They designed a high-throughput microwell array (up to 150,000 microwells) and isolated individual cells by gravity trapping. Then, polymer beads with attached oligo(dT) primers were trapped in the same microwell in order to obtain a cell-bead pair. Each bead contains sequences for amplification of captured mRNA as well as a unique barcode for identification. The team was able to successfully sequence RNA in a high-throughput manner with single-cell resolution. In 2015 Kimmerling et al.^41^, designed a microfluidic chip with single hydrodynamic traps to isolate individual CD8^+^ T cells. Through their design, they were able to trap and culture individual cells under defined conditions. The design enabled the capture of daughter cells from proliferating trapped cell. Single-cell release and recovery was performed and cells were analyzed by off-chip scRNA-seq. Thanks to this design, the team was able, by comparing intra- and inter-lineage expression of specific genes, to create a link between gene expression profiles and cell-division cycles. This type of platform has great potential for any studies on heterogeneous populations of cells with different clonal histories, as is the case for adaptive immune cells (B and T cells) and could be used to study the evolution of inflammatory states of a given clonal population and their progeny over time and under different stimulants.

### Proteomics and cell signaling

Although passive microfluidic systems have been used for RT-PCR, they are generally more popular for proteomics applications. As briefly mentioned above, the microengraving technique (Fig. 1b) is nicely suited for single-cell secretome screening. This process relies on sequestration of individual cells into microwells where their secretome can be captured by antibodies immobilized to the glass surface used to seal the microwells. Microengraving can effectively screen specific protein secretion down to the single-cell level. This process was pioneered by Love’s group and was used to actively study cytokine secretion profiles in lymphocytes^42–46^. For example, Varadarajan et al.^45^ in 2012 incubated mononuclear cell populations with a specific antigen, followed by single-cell isolation by microwell trapping. Microwells containing CD8^+^ T cells were identified and their pro-inflammatory cytokine profiles (IFN*γ*, IL-2, and TNF) were quantitatively analyzed (Fig. 1b). Cells showing increased pro-inflammatory cytokines, and therefore providing a potential effective immune response, can be retrieved through micromanipulation and expanded in vitro for further analysis. Their method was able to successfully establish clonal CD8^+^ T-cell lines with high specificity for HIV antigen in a rapid, cost-effective process while requiring 100- to 1000-times less cells than conventional methods and without the need of sequencing. Similar microengraving techniques were used on B cells to identify specific antibody-producing cells among a heterogeneous population^47–50^ and even on macrophages^51^. Microwells were also used to monitor long-term survival and proliferation rates of isolated T cells subjected to different culture conditions^52^.

### Cell–cell and cell–environment interactions

Another popular application of passive microfluidic systems is for cell–cell interaction studies. The ability to isolate pairs of cells in a defined environment and to monitor their interactions spatially and temporally is a key aspect in immune research. Immunotherapies are a new approach for cancer treatment and rely on using the immune system to fight tumors. A promising approach for immunotherapies is to isolate and enrich antigen-specific T cells and screen for the most potent ones, which show the highest anti-tumor activity. The process of T-cell activation is a complex mechanism involving the screening by T cells of potential antigen peptides. Although T cells (especially CD8^+^ T cells) can be screened for antigen presence in stromal cells (through T-cell receptor (TCR)–peptide major histocompatibility complex (pMHC) interactions), immune reactions often rely on phagocytic uptake of antigens by dendritic cells (DCs) (e.g. virus-infected cells ultimately dying and releasing viral antigens that are phagocytosed by DCs). The DCs will then display the antigen and travel to a lymph node where a high concentration of lymphocytes (B and T cells) are present. When a defined peptide antigen matches the specificity of a given T-cell clone, this clone will activate and expand in order to eliminate the antigen’s source. Therefore, the ability to understand and create DC-T or B-T cell interactions is of great importance in immunotherapies. However, each T-cell clone is genetically unique, and therefore such interactions should be handled at the single-cell level. Several research groups have used passive microfluidic systems to isolate individual T cells and match them with different cell types to monitor their activation and proliferation potential. Dura’s group^53,54^ created a microfluidic system with hydrodynamic traps able to capture pairs of immune cells (Fig. 1c). Their system uses specifically designed traps to capture single cells, transferring them into larger trapping areas where single cells from a second cell type can be loaded and trapped. Dura’s group and others used this system to profile lymphocyte interactions between an antigen-loaded B cell and a T cell^54,55^ but also between other types of immune cells such as natural killer (NK) cells^56^. Dura’s group was able to monitor early activation dynamics of single T cells and to study the heterogeneity of T-cell activation. Compared to conventional approaches, this platform offers the advantages of precise spatial and temporal control over single-cell pairs. This platform also allows control over cell culture conditions with the possibility of adding reagents to test different microenvironmental conditions without losing cell pairs and is well-suited for samples with low cell numbers, which are insufficient for analysis using conventional techniques such as flow cytometry.

After an encounter with its specific antigen, a T cell will proliferate and activate. Then, the T cell will migrate toward the source of antigen. T cells can follow inflammatory signal gradients throughout the body in a process called chemotaxis. T-cell migration is an important step and many immunotherapeutic approaches for cancer treatment aim to maximize T-cell chemotaxis. Passive microfluidic systems have been used to recreate inflammatory gradients to study how T cells migrate. In 2006, Lin et al.^57^ created a microfluidic device able to generate precise gradients of chemokines, both in time and space to study the migration of lymphocytes toward different chemokine gradients and were able to identify potent candidates (CCL19 and CXCL12). Another example of T-cell chemotaxis in a microfluidic device was performed by Jain et al.^58^ in 2015, where they created a microfluidic maze with pro-inflammatory cytokine gradients to study patterns between activated and unstimulated lymphocytes. In cancer research, passive microfluidic systems with gradient generators or with migration chambers are good tools to study immune response against tumor cells^59–62^.

## Active microfluidic systems

Although passive microfluidic systems provide significant advantages over conventional methods for single-cell applications, it is often difficult or impossible to perform complex fluidic manipulations. Certain applications require dynamic inputs during the experiment or testing of many different reagents or conditions in parallel. In 2000, Quake’s group^63^ developed a process called “multilayer soft lithography”, by bonding multiple patterned layers of PDMS elastomer together. PDMS and other elastomers are soft materials that can be deformed elastically with small actuation forces. This property can be used to fabricate on-chip valves by stacking microfluidic channels and pressurizing the control channel. In response to the pressure increase, a membrane separating the two channels will deflect and reversibly pinch off the flow channel. The actuation of such valves is a fast process and the response time is on the order of milliseconds^63^. By combining multiple valves, the group fabricated functional elements including peristaltic pumps and multiplexers. The use of valves enables precise flow control on-chip. By combining valves in specific manners, highly complex flow paths can be controlled with a relatively small number of control inputs^64^. Microfluidic Large-Scale Integration (mLSI) has become a useful tool in biological research. In this review we will focus only on their uses for single immune cell studies. Other applications and uses of active microfluidic systems can be found in several reviews^10,29–33^.

### Genomics

**Fig. 2:**
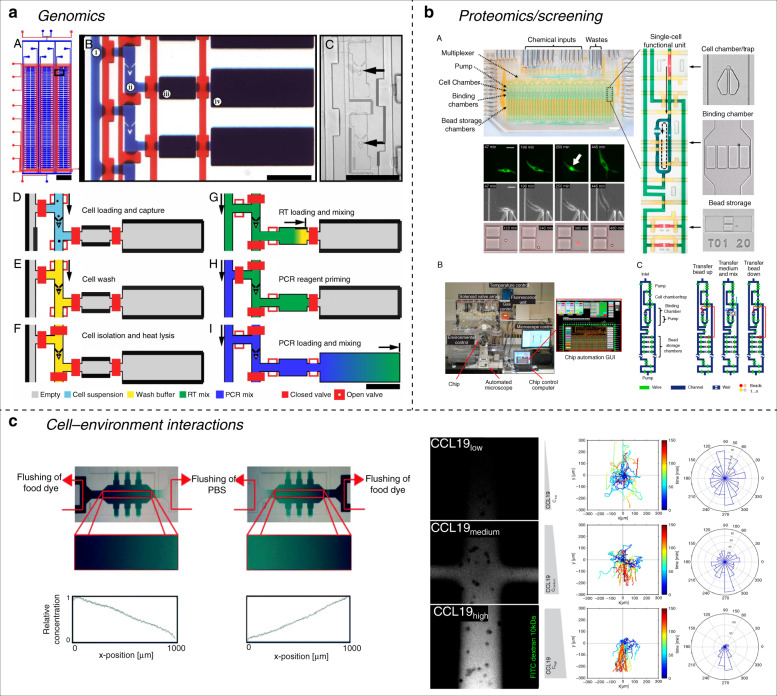
Active devices for single-cell analysis. **a** The use of valves allows compartmentalization of small volumes, which is an useful for performing on-chip single-cell RT-PCR. **b** Compartmentalization can be used to isolate single cells and study their secretion dynamics over time. **c** Active systems can precisely control flow patterns, enabling the creation of protein gradients in time and space. Immune cell migration toward different cytokine profiles can be studied at the single-cell level. Reprinted with permission^69,72,79,80^

Active microfluidic systems have been used for single-cell genomics for the past decade. Their ability to isolate small volumes (using valves) and to mix different reagents are useful functions for transcriptome analysis. The use of a ring mixer has been a popular method for single-cell RT-PCR^65–67^. A study even combined a microfabricated capillary electrophoresis separation channel for product analysis^68^. In 2011, White et al.^69^ designed a fully integrated single-cell RT-qPCR microfluidic platform performing on-chip cell capture, cell lysis, and reverse transcription followed by off-chip quantitative PCR (Fig. 2a). To achieve such designs the team combined the use of hydrodynamic traps to isolate single cells and the use of valves to create dedicated chambers for cell lysis, loading of reagents and mixing. This platform was applied to measure more than 3000 single-cell miRNA expression profiles. This type of platform has great potential for single immune cell genomics. By enabling isolation and transcriptome analysis of single T cells, such platforms could greatly enhance the study of immune cell heterogeneity. Although active microfluidic systems are well-suited for single-cell genomics, droplet microfluidics has become more popular and suitable as we discuss in the droplet microfluidics section.

### Proteomics and cell signaling

Compared to passive devices for proteomics such as microengraving, microfluidic devices involving active systems possess certain advantages. Using valves, such systems can isolate small volumes in which single cells are contained. The concentration of secreted molecules will increase over time and therefore the sensitivity of detection is higher. Several research groups have used active valve-based devices to study single-cell secretion. Ma et al.^70^ in 2011 created a microfluidic system containing arrays of different capture antibodies. Using valves, they achieved single-cell separation and measured, for each cell, more than 10 different proteins. Another approach with active valves was achieved in 2010 by Singhal et al.^71^, where they created a microfluidic device for studying antibody–antigen binding kinetics on beads and single cells. While elastomeric valves can, as described above, collapse the flow channel, their geometry can be adapted to produce sieve valves. A sieve valve is an actuated valve that does not fully collapse the lower channel but leaves a narrow opening on the sides (a leaky valve). This feature can be useful when cells need to be trapped and retained in a microchamber while different reagents need to be added to the cell culture condition. Singhal et al. used sieve valves to trap beads coated with antibodies to study association and dissociation rate measurements of antigens to their specific antibodies. The use of active systems enabled the ability to isolate beads and to control the fluidic operations required to study protein interaction kinetics. The group also focused on capturing antibodies produced by hybridoma cells by pairing a single hybridoma cell with antibody capture beads, followed by antigen loading. This technique facilitated single-cell screening of antibody-producing cells for a specific antigen by providing full spatial and temporal control over flow behavior, tracking of capture beads and antigen-producing cells using sieve valves and sensitive fluorescent readouts. Quite similarly, Tay’s group^72^ in 2016 used active microfluidic systems to create a platform combining nanoliter immunoassays, microfluidic input generation, and time-lapse microscopy to measure time-dependent cytokine secretion and transcription factor activity from single macrophage cells under different inputs. Their work is a sophisticated example of a fully automated, multiplexed platform capable of trapping and isolating single macrophages while detecting cytokine secretion with capture beads downstream. By using traps and valves, the device can trap and isolate single cells but also different types of cytokine-specific beads for multiple readouts. This system can manipulate single beads to sequentially expose them to the trapped cell secretome for cytokine capture in a controlled manner. In this study, Junkin and colleagues^73^ looked at the dynamics of TNF secretion and NK-*κ*B expression under foreign antigens (e.g. LPS). The same team further improved on their integrated platform by removing the use of antibody capture beads by the use of on on-chip antibody patterning developed previously by our group for large-scale protein interaction studies^74,75^ and molecular diagnostics^76,77^. This protocol allows the immobilization of antibodies to the chip surface using a MITOMI “button”^78^. MITOMI buttons were used to functionalize the chip surface for capture of antibodies at precise locations and allowed the multiplexing of antibody types. The platform by Tay et al. works as follows: a single macrophage cell is trapped via a hydrodynamic trap in a chamber. This chamber is then isolated via valve actuation and the macrophage, cultured with foreign antigens, will activate a pro-inflammatory transcription factor (NK-*κ*B) and secrete inflammatory cytokines (e.g. TNF) that can be both analyzed through the cytokine-specific, spotted antibodies and through the use of reporter genes for the transcription factor. These two studies are good examples of the advantages of active microfluidic systems. Because of active valve-based control, such systems can provide a fully integrated, large-scale analysis of cell secretion and proteomics while retaining single-cell resolution and limiting cross-contamination between cells.

### Cell–cell and cell–environment interactions

Like passive systems, active microfluidic systems are good candidates to study cell chemotaxis. As already discussed, chemokine gradients can be generated on-chip and T-cell migration toward certain chemokines can be recorded and quantitated. Active systems can provide better flexibility in flow and cell handling than passive systems. In 2015, Mehling et al.^79^ developed an automated microfluidic cell culture system with stable, diffusion-based chemokine gradients. Their device enabled the precise placement of cells in the migration chamber and single-cell displacement could be tracked by fluorescence microscopy. The chip also provided several outlets along the migration chamber to extract lymphocytes based on their migration speeds for further off-chip analysis. With this system, the team studied migration of individual human primary T cells toward a CXCL12 gradient^79^. In physiological conditions molecules that are involved in cellular locomotion are not only soluble, they can also be attached to the extracellular matrix (ECM). To recreate this complex network of bound and unbound chemokines, Tay’s group^80^ designed a microfluidic device generating a gradient of soluble chemokine through the use of active fluidic controls combined with patterned chemokine gradients immobilized to the chip surface. To achieve chemokine immobilization, they used a technique called Laser Assisted Protein Absorption by Photobleaching (LAPAP)^80^. They used their device to track DC migration toward bound CCL21 and soluble CCL19 chemokines.

Overall, in this section, we looked at how the implementation of active systems have boosted the use of microfluidics for single-cell applications. Active systems enabled precise spatial and temporal control of the flow within microfluidic chips. Large-scale integration enabled parallelization and increased the throughput of experiments. Single immune cell research took advantage of active microfluidic systems for controlling and analyzing cells in defined, dynamic microenvironments.

## Droplet microfluidics

In order to obtain information about a single cell and to study the heterogeneity of a cell population, confinement of single cells is essential. Advances in microfluidics and microengineering have enabled the confinement of single cells to picoliter-sized water droplets contained in an oil phase. Droplet generation frequency can be tuned and can vary from slow dripping to frequencies of several kHz^81^. Those droplets can encapsulate cells or biological materials, and therefore have the potential for high-throughput single-cell studies. Although water-in-oil droplets have been used since the 1950s, recent advances in droplet microfluidics have greatly increased their uses in single-cell analysis^82^. Our review is focused on droplet microfluidics for single immune cell applications, thus we will not describe the history and advances of this field extensively. We recommend our readers to read dedicated reviews covering droplet microfluidic technologies^82–84^. Here, we explore how droplet microfluidics has been used in research to tackle the various challenges that single-cell immunology presents. Examples of droplet microfluidics applied to genomics, proteomics, and cellular interactions are described below.

### Genomics

**Fig. 3:**
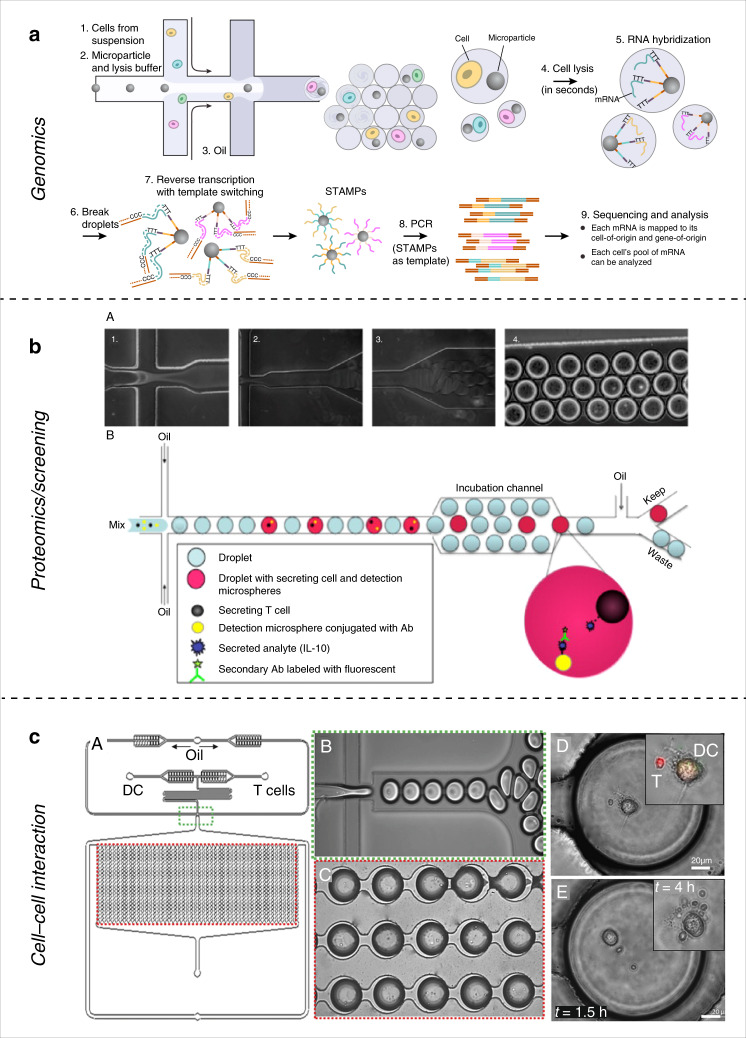
Droplet microfluidics for single-cell analysis. **a** Drop-seq technology allows for lymphocytes receptor sequencing and entire transcriptomes. Cells are co-encapsulated with lysis buffers and DNA-capture beads with unique barcode. **b** Single lymphocyte encapsulated in droplets containing cytokine-capture beads and secondary antibodies for cytokine secretion analysis. **c** Interactions at the single-cell level between antigen-presenting cell (DC) and a lymphocyte (T) can be recorded through droplet encapsulation of cell pairs. Reprinted with permission^87,92,97^

Single-cell RNA-sequencing (scRNA-seq) was achieved by Tang et al.^85^ in 2009. This technology allowed the study of the heterogeneity and diversity of gene expression among a cell population. However, using conventional techniques usually leads to low throughput for scRNA-seq. Moreover, certain studies require sequencing of rare samples with few cells. Droplet microfluidics enables the isolation of cells in picoliter-sized droplets, where appropriate chemistry can be added to achieve RNA-sequencing with high sensitivity. Recently, several groups achieved high-throughput scRNA-seq systems using commercial droplet technology, including inDrop^86^, Drop-seq^87^ (Fig. 3a) and the 10x systems^88^. All these systems are described and compared in a recent review by Zhang et al.^89^ Single-cell RNA-sequencing provides profiling of cellular mRNA expression and also enables analysis of the transcriptional state of single cells such as splice variants. Genome-wide expression profiles of single cells was achieved by Macosko et al.^87^ in 2015, with their Drop-seq platform. It allows for single-cell mRNA extension through PCR combined with molecular barcoding for single-cell tracing. They used their platform to analyze the transcriptome of almost 50,000 mouse retinal cells and were able to identify several transcriptionally different populations. One big advance in the field of single immune cell analysis was the sequencing of both immune receptor chains from millions of lymphocytes at the single-cell level by McDaniel et al.^90^ in 2016. They encapsulated cells with lysis reagents and magnetic beads in droplets. The lysed cells release their mRNA which are captured by the magnetic beads. Then emulsions are broken and the beads are separated, purified, and mixed with RT-PCR solutions with the final products submitted for sequencing. This platform allows a single researcher to achieve T-cell receptor (TCR) sequencing within a day^90^. The sequencing of variable domains of immune receptors (antibodies and TCRs) has enormous potential for cancer research and personalized medicine as a whole. Although the field of droplet microfluidics for single-cell sequencing, both genome-wide and RNA-sequencing, is quite young, it promises to improve our understanding of the immune repertoire at the single-cell level. Such technologies also have the potential to help identify rare immune cell populations and to develop personalized, immune cell-based therapies as potent new standards for complex diseases like cancer.

### Proteomics and cell signaling

As we discussed in the previous sections, secretion of cytokines by immune cells is a powerful readout. It provides information about the specificity and the function of immune cells. However, bulk experiments lack single-cell resolution and average the cytokine secretion of an entire immune population. Single-cell technologies aim to study the heterogeneity of immune cells. Single-cell cytokine secretion studies are important for isolation of rare, antigen-specific T cells and could lead to improved immunotherapies. As observed with active microfluidic systems, the confinement of single cells with capture beads for cytokine secretion are useful approaches. Droplet microfluidics offers the advantages of increasing the sensitivity and throughput of the experiments, while retaining isolated single cells in droplets. In 2013, Chokkalingam et al.^91^ presented a droplet microfluidic platform to detect cytokines (IL-2, IFN-*γ*, TNF-*α*) secreted from single, activated T cells over time. Their approach relies on the use of agarose-gel droplets encapsulating a single T cell with different cytokine-capture beads. During the incubation, the activated T cell will secrete cytokines that will be captured by the capture beads in the near vicinity within the droplet. The droplets can then be separated, washed and incubated with secondary fluorescent antibodies for quantification of cytokine presence and analyzed by flow cytometry^91^. Their method allows high-throughput detection of T-cell activation heterogeneity and allowed to define subsets of cell populations based on their function. Droplet microfluidics has the ability to isolate single cells in defined microenvironments, while retaining high-throughput. Similar approaches for cytokine detection in single T cells in droplets were designed^92–94^, such as the work of Konry et al.^92^ (Fig. 3b), where IL-10 cytokine secretion was studied in droplets containing single T cells. The group added the secondary antibody to the droplets to achieve, after incubation, on-chip sorting between cytokine producing cells and the rest. The same group used similar designs to study cytokine secretion in dendritic cells^93^. Also using droplet microfluidics, Qiu et al.^94^, designed a T-cell surface aptamer sensor for measuring cytokine secretion at the single-cell level through single-cell isolation in droplets. Their aptamer probe can be anchored onto the cell surface and upon cytokine binding to the aptamer, a fluorescent signal will be emitted that can be analyzed by fluorescence microscopy and flow cytometry. In addition to cytokine analysis, droplet microfluidics was used for single-cell phenotyping of antibody-secreting cells. Eyer et al.^95^ designed a droplet microfluidic system combined with a sandwich immunoassay for IgG phenotyping. They used nanoparticles pre-coated with antibodies to capture secreted immunoglobulin. Using a magnetic field, the nanoparticles can form an aggregate, observable by microscopy. Those are only a few examples where droplet microfluidics was used for proteomics applications for single immune cell research highlighting the advantages of this technology. Whether it is cytokine secretion from T cells or screening of antibody-producing B cells, droplet microfluidics provides a high-throughput, sensitive platform to study immune cell functions at the single-cell level.

### Cell–cell and cell–environment interactions

Droplet microfluidics can also be used to encapsulate cell pairs and study their interactions. This feature has great potential for single-cell immune analysis as it allows direct observation of immune cell interactions and their outcome on cell survival, proliferation and activation. Konry et al.^93,96,97^ encapsulated single pairs of T cells and dendritic cells into droplets, which were arranged into microarrays for monitoring of T-cell activation (Fig. 3c). Using this setup, they suggested that activation of single T cells is more rapid and efficient if T cells are in contact with DCs. Another example of encapsulating immune cells in droplets for cell–cell interaction studies is the work of Segaliny et al.^98^, where they trapped specific T cells with target cells (e.g. tumor cells) to monitor T-cell activation. T cells were engineered to express eGFP upon encounter with their specific antigen. Engineered T cells were then co-encapsulated into droplets with antigen-presenting cells. Then, the droplets were trapped using a microwell array for monitoring. The setup also allows for specific recovery of individual droplets. By heating the oil around a trapped droplet with a laser, a bubble forms and pushes the cell-containing droplet away from the trap and back into circulation. Recovered droplets can then be processed for TCR sequencing. The described setup provides a fully integrated solution for T-cell screening, isolation and recovery on the single-cell level and can be linked to downstream genomic analysis. The ability to isolate a single T cell based on its specificity for an antigen and the ability to sequence its TCR receptor to extract information about this specificity is important for cancer immunotherapy treatments. Although conventional bulk assays would require T-cell clonal expansion to reach sample numbers sufficient for analysis by flow cytometry, which is a time-consuming and expensive step, microfluidics can provide single-cell methods sensitive enough to eliminate the need of clonal expansion while retaining high-throughput. Although requiring some cell engineering beforehand, which can be difficult for some applications, Segaliny’s work demonstrates the potential of droplet microfluidics for immunological cell–cell interaction studies.

## Discussion

The immune system is very complex and involves the intertwined network of several cell types. In addition to complex interactions between cells, certain immune cells are genetically unique. The ability to separate and study cell behaviors in controlled environments is required to deepen our understanding of the immune system and help regulate cases where the immune system machinery is broken or ineffective, such as autoimmune diseases and cancer. The behavior of single immune cells is the result of highly complex networks that involve a delicate balance between intracellular and extracellular signaling. Immune cells can be considered as a black-box that integrates internal and external triggers into a proper response. In order to comprehend an immune cell response, the complex mechanisms that regulate an immune cell must be unraveled. Understanding single-cell specificity and dynamics require appropriate tools that conventional, population-based methods cannot deliver. Microfluidic devices have reached single-cell resolution and, because of their increased throughput, helped achieve multi-parameter measurements. Microfluidics enabled researchers to create controlled, dynamic microenvironments where single cells can be interrogated. Advances in different areas of immunology have been made because of microfluidic systems such as advances in single-cell genomics, proteomics, secretion dynamics and signaling as well as advances in cell interactions with their environment and between themselves.

However, due to the complexity of the immune system, much remains to be done. Immune cells are highly responsive to a multitude of complex signals from their environment, both in time and space. Experiments measuring only few parameters are often not adequate for deepening our understanding of how the immune system works. In order to further decipher the complexity of the immune system, multi-parameter experiments should be conducted on single cells in a precisely defined, dynamic microenvironment. Microfluidic systems have been used successfully to recreate complex environments for single-cell analysis and have enabled studies of single-cell dynamics from genomics, proteomics to cell–cell interactions.

The integration of multi-parameter experiments on single immune cells will enable the acquisition of large datasets that, through the use of machine learning and other artificial intelligence methods, train and create computer models of immune cells and immune responses^99^. We believe that microfluidics can play a key role in achieving that goal through automation. Integrated microfluidic systems can be automated through computer-controlled interfaces. By controlling a microfluidic chip, as well as data acquisition and analysis, automated microfluidic systems can create a closed-loop system with real-time data acquisition and analysis with potential decision-making abilities. Advances in machine learning and artificial intelligence will strengthen the potential of automated microfluidic systems and could lead to fully integrated measurement and screening devices. Microfluidics, however, is not without drawbacks. One weakness of microfluidic systems is reliability. Indeed, microsystems are often prone to clogging by dust or small debris. Intelligent systems must be developed to ensure reliability and robustness. Despite those weaknesses, we believe that microfluidic devices also will have an important role to play as point-of-care applications for immunology. Integration of microfluidic systems in clinical pipelines could help reduce time and cost for personalized treatments including immunotherapy. Personalized cancer treatments based on modified T cells with high anti-cancer specificity, either by immunoengineering CAR-T cell technologies^100^ or by isolating naturally occurring, rare anti-tumor T cells, are already under clinical trials, including immune checkpoint blockade (PD1-PD-L1) trials^101^ that involve T-cell engineering for immunotherapy, and show great promise for personalized cancer treatments. Immunotherapies present the advantages of greatly reducing the probability of tumor relapse by creating memory T cells. However, extensive, laborious, time-consuming and expensive protocols are currently needed to manipulate single T cells for personalized treatments. Clinics need more effective and high-throughput platforms for single immune cell genomics and proteomics to identify and address potential targets on a patient-to-patient basis. Microfluidic systems have the potential to cut the cost and analysis time required to achieve single-cell analyses as an integrated aspect of the clinical pipeline. Some start-ups have already successfully implemented microfluidic devices for single immune cell analysis such as the C1 system by Fluidigm, or the Chromium system by 10x Genomics. Given the rapid technological advances in the past 1–2 decades, it seems plausible that in the near future single-cell techniques are used routinely, from check-up diagnostics at the pharmacy to highly complex single-cell tools used in clinics for personalized treatments in the field of cancer or autoimmune diseases.
